# RNA Interference-Mediated Simultaneous Suppression of Seed Storage Proteins in Rice Grains

**DOI:** 10.3389/fpls.2016.01624

**Published:** 2016-10-31

**Authors:** Kyoungwon Cho, Hye-Jung Lee, Yeong-Min Jo, Sun-Hyung Lim, Randeep Rakwal, Jong-Yeol Lee, Young-Mi Kim

**Affiliations:** ^1^Rural Development Administration, Department of Agricultural Biotechnology, National Institute of Agricultural ScienceJeonju, South Korea; ^2^Faculty of Health and Sport Sciences and Tsukuba International Academy for Sport Studies, University of TsukubaTsukuba, Japan; ^3^Global Research Center for Innovative Life Science, Peptide Drug Innovation, School of Pharmacy and Pharmaceutical Sciences, Hoshi UniversityTokyo, Japan

**Keywords:** seed storage protein, glutelin, prolamin, globulin, suppression, RNAi

## Abstract

Seed storage proteins (SSPs) such as glutelin, prolamin, and globulin are abundant components in some of the most widely consumed food cereals in the world. Synthesized in the rough endoplasmic reticulum (ER), SSPs are translocated to the protein bodies. Prolamins are located at the spherical protein body I derived from the ER, whereas, glutelins and globulin are accumulated in the irregularly shaped protein bodies derived from vacuoles. Our previous studies have shown that the individual suppression of glutelins, 13-kDa prolamins and globulin caused the compensative accumulation of other SSPs. Herein, to investigate the phenotypic and molecular features of SSP deficiency transgenic rice plants suppressing all glutelins, prolamins, and globulin were generated using RNA interference (RNAi). The results revealed that glutelin A, cysteine-rich 13-kDa prolamin and globulin proteins were less accumulated but that glutelin B and ER chaperones, such as binding protein 1 (BiP1) and protein disulfide isomerase-like 1-1 (PDIL1-1), were highly accumulated at the transcript and protein levels in seeds of the transformants compared to those in the wild-type seeds. Further, the transcription of starch synthesis-related genes was reduced in immature seeds at 2 weeks after flowering, and the starch granules were loosely packaged with various sphere sizes in seed endosperms of the transformants, resulting in a floury phenotype. Interestingly, the rates of sprouting and reducing sugar accumulation during germination were found to be delayed in the transformants compared to the wild-type. In all, our results provide new insight into the role of SSPs in the formation of intracellular organelles and in germination.

## Introduction

Seed storage proteins (SSPs) are one of abundant components (approximately 10–12%) in the most widely consumed cereals in the world, including the cereals wheat, maize, and rice. The SSPs are used as a nitrogen source for seedling development and as a nutrient source for human and livestock consumption (Kawakatsu et al., 2010). According to the solubility in the extraction solvents, SSPs are classified as water-soluble albumins, saline-soluble globulins, alcohol-soluble prolamins, and acid/alkaline-soluble glutelins (Kawakatsu et al., 2008; Saito et al., 2012).

Unlike the major cereals, such as barley, maize, and wheat where the major SSP is prolamin, glutelins in rice seeds account for 60–80% of the total SSPs. Rice glutelins are encoded by 12 genes excluding three pseudo-genes and classified into four groups (GluA, GluB, GluC, and GluD) according to amino acid sequence similarities (Kawakatsu et al., 2008). Glutelins are synthesized as a precursor protein (proglutelin) in the endoplasmic reticulum (ER) and transported to protein body II (PB-II) storage vacuoles (PSVs) via the Golgi apparatus. They are ultimately processed into mature 37-kDa acidic and 20-kDa basic subunits interlinked by disulfide bonds (Tanaka et al., 1980; Yamagata and Tanaka, 1986). Rice prolamins, which account for 20–30% of the SSPs, are encoded by 34 genes and classified into three groups (10, 13, and 16-kDa prolamins) according to their relative molecular weights. Based on the number of cysteine (Cys) residues, the 13-kDa prolamins are further classified into four groups such as Cys-rich prolamin 13a-I and -II and Cys-poor prolamin 13b-I and -II (Saito et al., 2012). Prolamin synthesis is initiated in the ER, and it accumulates in the ER lumen, where it forms spherical protein bodies (PBs; PB-I) (Tanaka et al., 1980; Yamagata and Tanaka, 1986). Globulin accounts for 2–8% of the SSPs in rice and is encoded by a single gene. Globulin is synthesized as a 21-kDa precursor and is then transported to PSVs (PB-II) via the glutelin transport system, where it is processed into a 19-kDa mature form (Tanaka et al., 1980; Yamagata and Tanaka, 1986; Kim et al., 2013).

Recently, it has been suggested that the seed is a good platform for producing valuable recombinant proteins due to multiple advantages, such as high productivity, long-term storage stability, and low-cost oral delivery of therapeutic proteins (Takaiwa et al., 2015). However, as a downside, the accumulation of some recombinant proteins in the endosperm of transgenic rice seeds results in chalky and shriveled phenotypes. This is due to heavy loading on the ER lumen, termed ER-stress, and is accompanied by the expression of stress-related proteins. Several studies have shown that ER-stress induces protein-folding catalysis-related genes encoding binding protein (BiP), protein disulfide isomerase (PDI), and calnexin (CNX) (Rowling and Freedman, 1993; Kaufman, 1999; Kleizen and Braakman, 2004; Oono et al., 2010; Satoh-Cruz et al., 2010). BiP is a major ER chaperone that belongs to the HSP70 family and has an ATPase at its N-terminus and a protein-binding domain at its C-terminus, thus allowing BiP to interact with immature proteins on the ER lumen assisting in protein folding (Takaiwa et al., 2009; Wakasa et al., 2011). PDI catalyzes the formation and rearrangement of disulfide bonds, and CNX selectively binds to the unfolded glycoproteins and prevents the transport of misfolded proteins from the ER to the Golgi apparatus (Kleizen and Braakman, 2004). Numerous studies have reported that gain/loss of functions of the ER chaperones and proteins implicated in the SSP secretory system induce ER stress, leading to changes in the SSP levels and intracellular structure within the cells of seed endosperms producing chalky and shriveled seeds (Barlowe, 2002; Takemoto et al., 2002; Kleizen and Braakman, 2004; Takaiwa et al., 2009; Satoh-Cruz et al., 2010; Wakasa et al., 2011; Tian et al., 2013).

Maize *opaque2* (a basic leucine zipper transcription factor) mutant showed a floury kernel phenotype with an increased lysine content and a reduction in the 22-kDa α-zein similar to rice 10-kDa prolamin (Schmidt et al., 1990; Segal et al., 2003). RNA interference (RNAi)-mediated suppression of α-zein resulted in the floury kernel phenotype (Wu and Messing, 2010). To improve the nutrition quality, several groups have performed studies on the molecular and cellular features of SSP-deficient rice and maize seeds (Kawakatsu et al., 2010; Wu and Messing, 2010; Kim et al., 2013; Lee et al., 2015). In the maize prolamin (known as zein)-suppressed mutants, reduction of α-zein (similar to rice 10-kDa prolamin) resulted in an increased level of lysine. Moreover, the suppression of α-zein and the simultaneous reduction of both β- and γ-zein led to the formation of abnormal PBs and incomplete embedding of starch granules, resulting in floury kernel phenotype (Wu and Messing, 2010). In SSP-repressed rice transgenic seeds, the suppression of individual SSPs resulted in the increased expression of other SSPs and structural changes in the PBs of endosperm cells without severe seed-phenotypic changes (Kawakatsu et al., 2010; Kim et al., 2012, 2013; Lee et al., 2015).

Recently, several research groups reported that the accumulation yields of recombinant proteins are increased in transgenic rice seeds suppressing prolamins (prolamin 13b and 10-/16-kDa Pro) or glutelins (GluA and B) compared to their yield in wild-type rice, suggesting the utilization of SSP-suppressed transformants to improve the nutrient quality and recombinant protein yield (Kim et al., 2012; Yang et al., 2012). Previously, to gain a better understanding of the SSP-regulation system in rice seeds, we studied several transgenic rice plants suppressing respective glutelins, prolamins, and globulin in their seeds (Kim et al., 2012, 2013; Lee et al., 2015). Herein, to gain new insight into the simultaneous suppression of SSPs accumulated in PB-I and PSV of rice seed endosperms, we first generated SSP-suppressed transgenic rice plants that block the simultaneous expression of SSPs grouped in glutelin A (GluA), prolamin 13a-I and globulin using RNA interference approach (defined as GPGb-RNAi). Our results revealed that the massive suppression of three different types of SSPs induced morphological changes in the PBs and was strongly correlated with increases in the ER chaperones, including BiP1 and PDIL1-1, resulting in the reduction of starch contents and the phenotypic change of starchy granules in rice seed endosperms.

## Materials and Methods

### Construction of Binary Vectors and Rice Transformation

**FIGURE 1:**
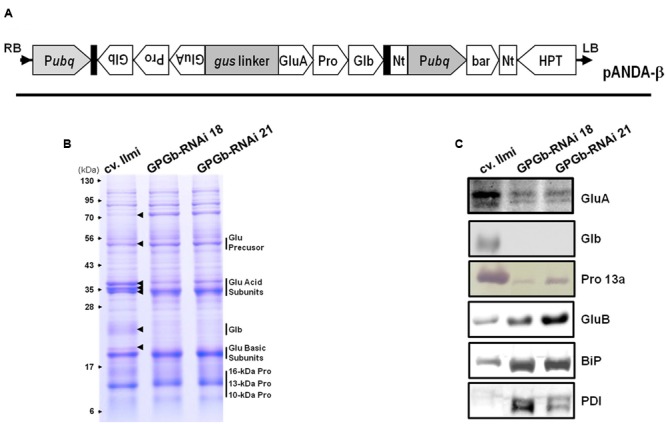
**Binary vector construct and expression analysis of seed storage proteins (SSPs) in the seeds of GPGb-RNAi rice transformants. (A)** Vector constructs for the simultaneous suppression of glutelin, prolamin, and globulin: P*ubq*, maize ubiquitin1 promoter; Nt, NOS terminator; bar, Bialaphos resistance gene; HPT, Hygromycin resistance gene. **(B,C)** Gradient SDS-PAGE (8–17.5%) and Western blot analyses using anti-SSP antibodies with total seed protein extracts derived from wild-type (cv. limi) and two independent GPGb-RNAi transgenic rice seeds (GPGb-RNAi 18 and 21). The arrows represent protein bands with different intensities among wild-type and the GPGb-RNAi seeds. GluA, glutelin A; GluB, glutelin B; Glb, globulin; Pro 13a, prolamin 13a; BiP, binding protein; PDI, protein disulfide isomerase.

To construct an RNAi cassette for suppressing storage proteins in rice seed including glutelin, prolamin and globulin, their conserved regions were cloned from *glutelin A-2* (*GluA-2*, Os10g0400200), *prolamin 13.2* (*Pro13.2*, Os07g0206500) and *globulin* (Os05g0499100) genes and linked as described in Supplementary Figure S1. The linked cDNA was amplified by PCR using a primer pair (F primer 5′-AAAAAGCAGGCTCAGTTTGCTTGTTCCTCT-3′ and R primer 5′-AGAAAGCTGGGTCTCGCCCTGGTCAGC-3′) containing an attB1 or attB2 sequence to allow the Gateway cloning system to be used. The amplified products were sub-cloned into the pDONR221 vector (Invitrogen, Carlsbad, CA, USA) using a recombination reaction. The RNAi cassette was recombined with the destination vector pANDA-β containing the maize ubiquitin 1 promoter, the napaline synthase (NOS) terminator and the bialaphose resistance gene (**Figure 1A**). The constructed binary vector was introduced to *Agrobacterium tumefaciens* (LBA4404), and the gene of interest was introduced into the rice calli induced from Japonica-type Korean rice cv. *Ilmi* using *Agrobacterium*-mediated transformation. Rice transformation was performed as previously described (Kim et al., 2012).

### Total Seed Protein Extraction, SDS-PAGE, and Immunoblotting

Mature dry seeds from independent transgenic rice plants were harvested, de-husked and ground to a fine powder using a mortar and pestle at room temperature. The total seed protein was extracted from the powder (100 mg) with 1 ml of sodium dodecyl sulfate (SDS)-urea buffer [250 mM Tris-HCl, pH 6.8, 4% SDS, 8 M urea, 20% glycerol, and 5% β-mercaptoethanol (2-ME)] and quantified using a protein assay kit (Invitrogen) as previously described (Kim et al., 2013; Lee et al., 2015). The total seed protein (10 μg) was separated by gradient SDS (8–17.5%) polyacrylamide gel electrophoresis (Saito et al., 2012). Following gel electrophoresis, the separated proteins were visualized by CBB-R250 staining or transferred to PVDF membranes (Millipore) for Western blot analysis. Total seed proteins from wild-type (cv. *Ilmi*) and GPGb-RNAi transformants were extracted from three independent experiments and then analyzed by SDS-PAGE, Western blot analysis and two-dimensional gel electrophoresis (2-DGE).

### Antibodies

Antibodies against GluA, GluB, and globulin were prepared previously in our laboratory (Kim et al., 2013; Lee et al., 2015). Antibodies to prolamin 13a and the BiP and PDI were provided by Dr. B. S. Han (Rural Development Administration, South Korea) and Prof. H. S. Seo (Seoul National University, South Korea), respectively. The primary antibodies against GluA, GluB, and globulin were produced in adult rabbits and diluted 1:2000. The primary antibodies against the prolamin 13a in rats and against the BiP and PDI in rabbits were diluted by approximately 1:2500 and 1:3000, respectively. The HRP- or AP-conjugated secondary antibodies (1: 10000) were purchased from Promega.

### Two-Dimensional Gel Electrophoresis

2-DGE was carried out using pre-cast IPG strip gels (Immobiline DryStrip pH 3-11 NL, GE Healthcare, Uppsala, Sweden) on an IPGphor3 unit (GE Healthcare Bio-Sciences AB, Uppsala, Sweden), followed by second dimension SDS-PAGE on a Protein II xi Cell electrophoresis (Bio-Rad). The total seed protein extracts in wild-type (cv. *Ilmi*) and GPGb-RNAi 18 transgenic line were precipitated in 15% TCA overnight and dissolved in 32.5 mM CHAPS rehydration buffer containing 18.2 mM dithiothreitol (DTT). The volume carrying 50 (or 100) μg total protein was mixed with 32.5 mM CHAPS rehydration buffer containing 0.5% (v/v) pH 3-11 IPG buffer to a final volume of 350 μL, loaded onto an 18-cm IPG strip (pH: 3–11) and rehydrated in-gel for 15 h at 20°C. IEF was carried out for a total of 80 kVh. The IPG gel strips were equilibrated with 75 mM Tris-HCl (pH 8.8) containing 6 M urea, 30% glycerol, 2% SDS and 1% DTT and then incubated with 75 mM Tris-HCl (pH 8.8) containing 6 M urea, 30% glycerol, 2% SDS, and 2.5% idoacetamide for 15 min. The second dimension SDS-PAGE was performed using a 12.5% gel and stained with Coomassie Brilliant Blue R-250 for 3 h followed by destaining with 10% methanol and 10% acetic acid. To select and quantify the differentially expressed protein spots in both wild-type and GPGb-RNAi transgenic rice seeds, 2-DGE was thrice biologically replicated. After scanning all 2-D gels, the matching and normalized volumes of the selected spots across the gels were analyzed using Image Mater Platinum 7.0 (GE Healthcare Life Sciences, USA). Statistically significant differences in the average normalized volumes of each spot between wild-type and GPGb-RNAi transgenic rice line were tested using independent Student’s *t*-test.

### Liquid Chromatography–Tandem Mass Spectrometry

The differentially expressed spots in the gels were excised and cut out into small pieces. In-gel digestion and a mass spectrometric (LC–MS/MS) analysis of the spots were performed at the National Instrumentation Center for Environmental Management (Seoul National University, South Korea) as follows. The gel pieces were dehydrated with acetonitrile until the gel pieces turned opaque white and then dried in a vacuum centrifuge for 30 min (Savant Speed-Vac, Holbrook, NY, USA). Enzymatic in-gel digestion of proteins was performed with trypsin in 50 mM ammonium bicarbonate overnight at 37°C. The tryptic digests were extracted with 50% acetonitrile with 5% formic acid, dried under vacuum and resolved in 50% acetonitrile with 5% formic acid (Imai and Mische, 1999). The tryptic peptide mixtures were analyzed by LC-nano ESI-MS/MS system consisting of Dionex U 3000 RSLCnano HPLC system and Thermo Scientific Q Exactive Hybrid Quadrupole-Orbitrap mass spectrometer (Thermo Scientific, USA) equipped with a nano-electrospray ionization source and fitted with a fused silica emitter tip (New Objective, Woburn, MA, USA). The peptide samples were injected in solvent A consisting of water/acetonitrile (98:2 v/v) with 0.1% formic acid, trapped on a Acclaim PepMap 100 trap column (100 μm × 2 cm, nanoViper C18, 5 μm, 100 Å, Thermo Scientific), washed for 6 min with solvent A at a flow rate of 4 μl/min, and then separated on a Acclaim PepMap 100 capillary column (75 μm × 15 cm, nanoViper C18, 3 μm, 100 Å, Thermo Scientific) at a flow rate of 300 nl/min. The LC gradient was run at 2–35% solvent B consisting of acetonitrile/water (98:2 v/v) with 0.1% formic acid over 30 min, then from 35 to 90% over 10 min, followed by 90% solvent B for 5 min, and finally 5% solvent B for 15 min. The eluted peptides were electrosprayed through a coated silica tip (PicoTip emitter, New Objective) at an ion spray voltage of 2,000 eV. Mass raw files were analyzed by using proteome discoverer 1.3 (Thermo Scientific, USA) with the NCBI *O. sativa* database to identify the proteins with a least two distinct peptides. Tryptic peptide identifications were obtained by using SEQUEST XCorr scores (≥1.5 for +1 charge state, ≥2.0 for +2, ≥2.25 for +3, and ≥2.5 for ≥ +4) and allowing two missed tryptic cleavage sites.

### Profiling Transcripts for the SSP, ER Chaperone, and Starch Synthesis Using qRT-PCR

Immature developing seeds from independent transgenic rice lines were harvested 2-weeks after flowering (WAF), de-husked and then ground to a fine powder using a mortar and pestle. The powder (100 mg) was used to extract the total RNA according to the manufacturer’s instructions using the RNeasy Plant Mini Kit (Qiagen, Germantown, MD, USA). The total RNA was extracted from three independent experiments. The RNA quality, yield, and purity were determined spectrophotometrically (NanoDrop 1000 spectrophotometer, Thermo Scientific, USA). The total RNA samples were first treated with RNase-free DNase (Stratagene, Agilent Technologies, La Jolla, CA, USA). First-strand cDNA was synthesized in a 20 μl reaction mixture with a SuperScript III First-Strand Synthesis System Kit (Invitrogen) using 1 μg total RNA according to the manufacturer’s procedures. To check the quality of the synthesized cDNA, RT-PCR was performed on the ubiqutin (Os02g0161900) gene. The synthesized cDNA was increased to a volume of 50 μl using the sterile water supplied in the kit. The reaction mixture contained 0.5 μl of the first-strand cDNA, 10 pmols of each primer set, and 12.5 μl of the AccuPower^®^ Greenstar qPCR Master Mix (2X) (Bioneer, Seoul, South Korea) in a total volume of 25 μl. The thermal-cycling parameters of the CFX96^®^ Real-Time Detection System (Bio-Rad) were as follows: after an initial denaturation at 95°C for 15 min, samples were subjected to a cycling regime of 40 cycles at 95°C for 10 s, 55°C for 10 s, and 72°C for 30 s, followed by a gradient from 65 to 95°C to produce the melting curve. The primer sequences used for the qRT-PCR are listed in Supplementary Table S2. The expression level of each gene was normalized using a housekeeping gene, ubiquitin (Os02g0161900) and then its relative expression level in the GPGb-RNAi transformants and wild-type was represented as log_2_ value of average fold change calculated from three biological replicates using Bio-Rad CFX Manager Software (ver. 3.0, Bio-Rad).

### Seed Germination Test

Mature dry seeds (100 seeds) harvested from wild-type (cv. *Ilmi*) and GPGb-RNAi transformants were sterilized with diluted hypochlorite (2%) for 30 min and rinsed with distilled H_2_O three times for 10 min. The sterilized seeds were soaked in a sterile Petri dish plated with two sterilized Advantec Filter Papers (No. 6) and soaked with 10 ml distilled H_2_O. Seeds were incubated in a clean growth room (light/16 h and dark/8 h) at 25°C for 6 days. Sprouted seeds were counted and sampled every day and were then stored at -80°C. The test was independently replicated three times and then the replicated samples were utilized to measure the contents of starch and reducing sugar.

### Starch and Reducing Sugar Content

Starch content in grain powders (100 mg) of GPGb-RNAi transformants were measured using a starch assay kit (Megazyme) according to the manufacturer’s procedures. To analyze reducing sugar contents, mature dry seeds and germinated seeds were de-husked, ground to fine powders, and dried in a dry oven at 60°C overnight. Each powder (80 mg) was resuspended in 80% ethanol (1 ml) using sonication for 10 min and vortexing at 80°C and 300 *g* for 1 h. The supernatant after centrifugation at 20,000 *g* at 4°C for 10 min was collected. Reducing sugars in the collected supernatant were then measured as previously described (Jeong et al., 2010).

### Microscopic Analysis

Immature seeds from wild-type and transformants at the two-WAFs were harvested, and fixed overnight in 1.25% glutaraldehyde and 2% paraformaldehyde in 50 mM PBS at 4°C as previously described (Kim et al., 2013; Lee et al., 2015). After the fixed specimens were washed in PBS, dehydrated in a graded series (to 50% from 100%) of ethanol and embedded in Epon 812, the specimens were sliced to ultrathin sections with an ultramicrotome (Leica). Sections were stained with a solution of uranyl acetate and lead citrate and observed with Transmission Electron Microscopy (TEM; LEO912AB, Carl Zeiss, Jena, Germany). To observe the morphological changes of the mature seeds, germinated seeds and starch granules, the seeds were transversely and vertically sectioned. Photographs of the samples were taken using a light microscope and a scanning electron microscope (SEM).

## Results

### Selection of GPGb-RNAi Transformants Suppressing Glutelin, Prolamin, and Globulin in Rice Seeds

Previously, we had generated RNAi transgenic rice plants suppressing glutelin, prolamin, and globulin, respectively, and examined the effects of their individual suppression on the nutritional quality and formation of subcellular organelles (Kim et al., 2012, 2013; Lee et al., 2015). Herein, to further our knowledge on the role of the SSPs, the simultaneous suppression of glutelin, prolamin, and globulin in rice seeds was performed. For this, the conserved regions of each gene family were linked (Supplementary Figure S1) and inserted into the genomic DNA of Japonica-type Korean rice cv. *Ilmi* using a binary vector system (**Figure 1A**), generating 35 independent GPGb-RNAi transgenic lines. Among these, seven lines survived to the next generation (T_1_). The GPGb-RNAi transgenic lines suppressing glutelin, prolamin and globulin were screened by gradient SDS-PAGE analysis. Two representative homozygous lines (GPGb-RNAi 18 and 21) were selected from the progeny (T_4_ generation) of the transgenic lines (**Figure 1B**).

In rice, glutelin, prolamin, and globulin are known to be encoded by 12 genes excluding three pseudo-genes (Kawakatsu et al., 2008), 34 genes (Saito et al., 2012), and a single gene (Lee et al., 2015), respectively. Phylogenetic tree analysis based on nucleotide sequence similarities showed that the cDNA fragment cloned from *GluA-2* had high similarity with *GluA-1, -2*, and -*3* genes, grouped to GluA, and that the cDNA fragment from *Pro13a.2* also had high similarity with *pro13a.1* and *pro13a.2* genes, grouped to prolamin 13a-I (Saito et al., 2012), as shown in Supplementary Figure S2. This suggested that glutelins and prolamins within GluA and prolamin 13a-I groups could be co-suppressed in the GPGb-RNAi transformants.

**Figure 1B** showed that the intensities of about 50-kDa glutelin precursors, the 35-kDa acid subunits and the 19-kDa basic subunits were changed in GPGb-RNAi transgenic rice seeds compared to those of the wild-type (cv. *Ilmi*). Previously, Kawakatsu et al. (2010) reported that six glutelin acidic subunits in rice seeds (from top to bottom; GluB-4, GluA-2, GluA-1, GluA-3, GluB-2, and GluB-1) can be detected in the CBB-stained SDS-PAGE gels. Consistent with their analysis, the three bands at 35-kDa in **Figure 1B** represent the GluB-4/GluA-2, GluA-1/-3, and GluB-2/-1 (from top to bottom). **Figure 1B** suggests that the GluA proteins are decreased while the GluB proteins are increased in GPGb-RNAi transgenic lines. Indeed, the suppression of two GluA bands and enhancement of one GluB band was confirmed by Western blot analysis (**Figure 1C**). Additionally, one band for the 21-kDa globulin precursor in the wild-type (cv. *Ilmi*) line could not be detected in GPGb-RNAi transgenic lines by SDS-PAGE and Western blot analysis. In case of prolamins, a change of 10-, 13-, and 16-kDa prolamins levels could not be observed through SDS-PAGE. However, Western analysis revealed that the prolamin 13a (Cys-rich 13-kDa prolamins) in the GPGb-RNAi transgenic lines is significantly suppressed compared to the wild-type lines, indicating that other 13-kDa prolamin isoforms grouped to prolamin 13b might be increased in the GPGb-RNAi lines. Taken together, the results indicate that the GluA, prolamin 13a and globulin proteins are simultaneously suppressed in the seeds of the GPGb-RNAi transgenic lines.

### Differentially Expressed Protein Profiling in Seeds of GPGb-RNAi Transgenic Rice Using 2-DGE

**FIGURE 2:**
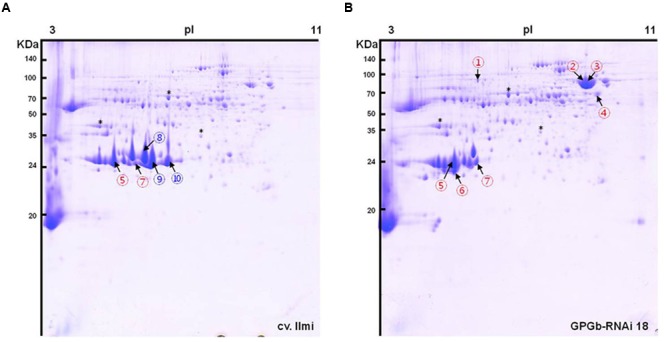
**Two-dimensional gel electrophoresis (2-DGE) of total seed protein extracts derived from wild-type and GPGb-RNAi transgenic rice seeds.** A total of 10 spots were selected as differentially expressed protein spots between the wild-type **(A)** and GPGb-RNAi 18 transgenic **(B)** rice seeds. Red and blue numbers indicate the increased and decreased spots in the GPGb-RNAi 18 transgenic rice seeds, respectively. Asterisks denote the same position indicator between the two 2-D gels.

One band of approximately 70-kDa on the gradient SDS-PAGE gel was observed to be strongly increased in the GPGb-RNAi transgenic lines (**Figure 1B**). The average contents of total proteins extracted from one grain were similar between wild-type and transgenic lines (data not shown), indicating that the observed change is caused by the simultaneous suppression of SSPs. Furthermore to identify which proteins are differentially expressed in the GPGb-RNAi transgenic rice seeds, 2-DGE was independently performed three times in wild-type and GPGb-RNAi 18 lines as shown in **Figure 2** and Supplementary Figure S3. Protein spots (10 spots) that were differentially expressed in the GPGb-RNAi transgenic line compared to the wild-type were selected and identified using LC–MS/MS analysis (**Table 1**; Supplementary Table S1). The increased spots 1, 2, 3, and 4 in the GPGb-RNAi transgenic line were identified as membrane protein (Os03g0271200), putative chaperone binding protein (BiP1, Os02g0115900) and protein disulfide isomerase-like 1-1 (PDIL1-1, Os11g0199200). Indeed, Western blot analysis showed that BiP and two PDI bands are increased in the GPGb-RNAi transgenic lines (**Figure 1C**). Furthermore, spots 5, 6, and 7 were observed to be increased in the GPGb-RNAi transgenic lines and were identified as GluB-1, B-5, and B-2, respectively. Interestingly, the other three spots (spots 8, 9, and 10) were decreased 13.33, 15.00, and 75.20 fold, respectively, in the GPGb-RNAi transgenic line and identified as GluA family (GluA-1, A-2, and A-3).

**Table 1:** Identification of the differentially expressed protein spots in the GPGb-RNAi 18 transgenic rice seeds compared to the wild-type by LC–MS/MS analysis.

Spot #	Gene ID	Description	# of unique peptides	Coverage (%)	Average fold change	*p*-value
Spot 1	Os03g0271200	Chloroplastic outer envelope membrane protein precursor	6	17.40	1.92	0.008
Spot 2	Os02g0115900	Putative chaperone BiP	26	51.43	4.33	0.046
Spot 3	Os02g0115900	Putative chaperone BiP	23	47.52	3.48	0.006
Spot 4	Os11g0199200	Protein disulfide isomerase-like 1-1 (PDIL1-1)	10	24.80	3.44	0.037
Spot 5	Os02g0249800	Glutelin type-B 1	3	23.27	1.43	0.042
Spot 6	Os02g0268100	Glutelin type-B 5	8	44.87	21.49	0.003
Spot 7	Os02g0249600	Glutelin type-B 2	6	12.79	2.00	0.011
Spot 8	Os10g0400200	Glutelin type-A 2	2	27.91	0.07	0.004
Spot 9	Os01g0762500	Glutelin type-A 1	7	26.85	0.07	0.005
Spot 10	Os03g0427300	Glutelin type-A 3	6	25.17	0.01	0.007

### Grain Morphology of the GPGb-RNAi Transgenic Rice

**FIGURE 3:**
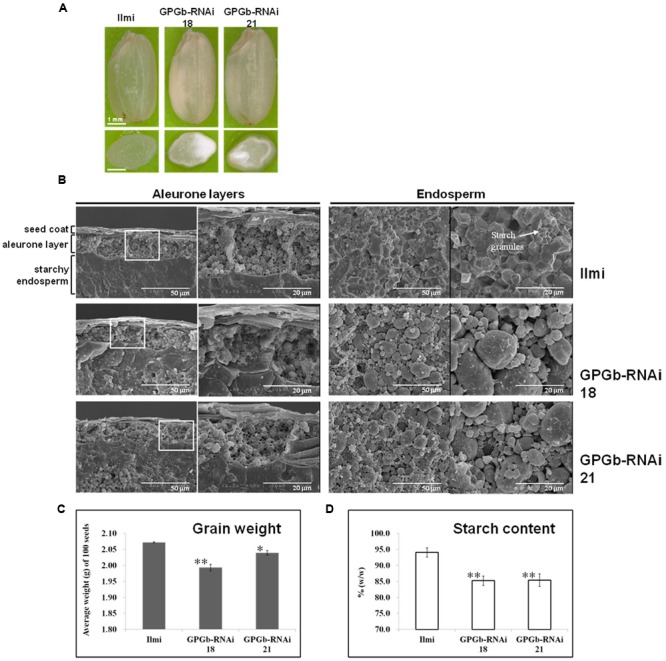
**Phenotype analysis of GPGb-RNAi transgenic rice seeds. (A)** Morphologies of the GPGb-RNAi 18 and 21 transgenic rice seeds and their transverse sections. **(B)** SEM images of aleurone layers and endosperms of wild-type (cv. *Ilmi*) and the GPGb-RNAi seeds. **(C)** Average dry weight (g/100 seeds). **(D)** Starch contents (% w/w). Significant differences of average dry weight and starch contents between wild-type and GPGb-RNAi transgenic rice lines were tested using independent Student’s *t*-test. ^∗^*p* < 0.1 and ^∗∗^*p* < 0.01.

Seeds of the GPGb-RNAi transgenic rice lines, which showed a clear suppression in the GluA, prolamin 13a and globulin proteins, were selected. Their endosperms were transversely and/or vertically sectioned. Sectioned specimens of the wild-type (cv. *Ilmi*) lines were virtuously lucid, whereas those of GPGb-RNAi were opaque with a floury feature (**Figure 3A**). SEM analysis showed that starch granules in seed endosperms of the wild-type cultivar (cv. *Ilmi*) are tightly packed in a polyhedral shape but those in GPGb-RNAi transgenic rice are instead loosely packed with diverse sphere sizes (**Figure 3B**). The morphological changes in the starch granules might be the reason behind the opaque and floury features in seeds of the GPGb-RNAi transgene rice. Furthermore, it was observed that the average weight of 100 grains and the starch content in the seed endosperms are lower in the GPGb-RNAi transgenic rice than in the wild-type cultivar (cv. *Ilmi*).

### Profiling Transcripts Implicated in SSPs, ER Stress, and Starch Synthesis Using RT-PCR

The combination of SDS-PAGE and 2-DGE along with microscopic analysis revealed the suppression of SSP expression, the enhanced accumulation of ER-chaperones and the morphologic changes of starch granules in GPGb-RNAi transgenic rice seeds. Furthermore, to observe the expression of the relevant genes, quantitative RT-PCR analysis was carried out in the immature seeds of the GPGb-RNAi transgenic rice at 2-week after flowering (WAF).

**FIGURE 4:**
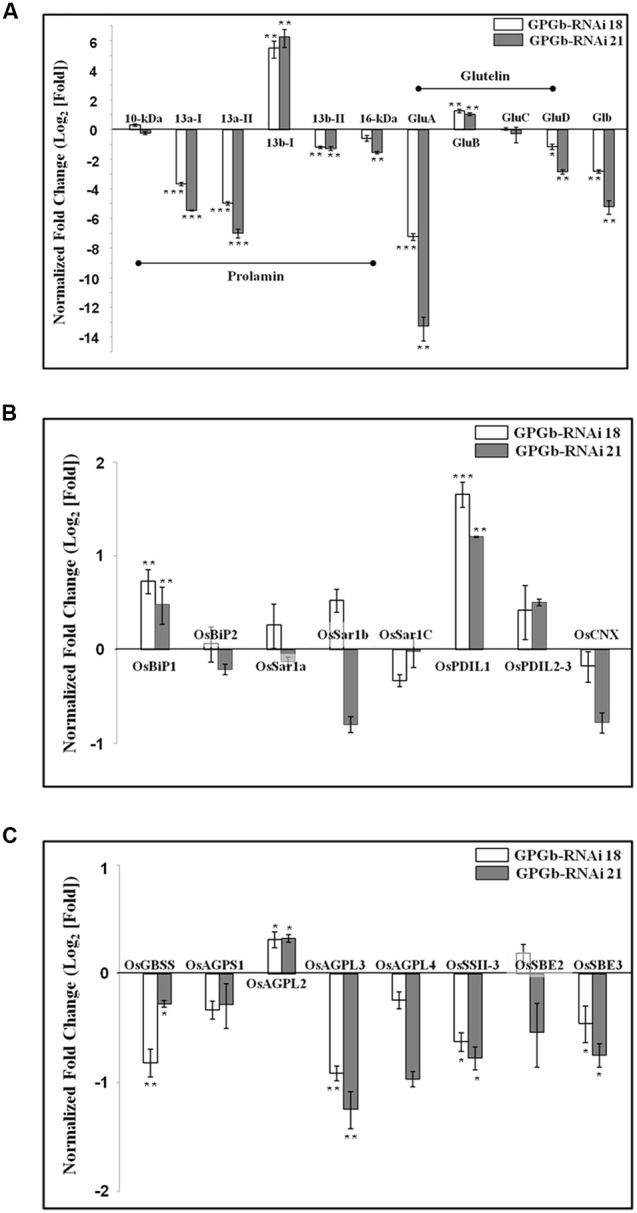
**Quantitative real-time PCR analysis in immature seeds of the GPGb-RNAi transformants.** The expression levels of seed storage proteins (SSPs) **(A)**, ER stress-related **(B)** and starch synthesis-related genes **(C)** were analyzed using total RNAs extracted from immature seeds of wild-type and GPGb-RNAi 18 and 21 transgenic lines at 2-week after flowering (WAF). After normalizing expression levels of target genes based on a reference gene, UBI, in each line, the relative level of individual gene in each GPGb-RNAi transgenic line versus wild-type is represented as log_2_ (average fold change) value. Error bars indicate the standard deviation (SD) and significant differences between means of each transcript level in wild-type and GPGb-RNAi transgenic lines were tested using independent Student’s *t*-test (^∗^*p* < 0.1, ^∗∗^*p* < 0.01, and ^∗∗∗^*p* < 0.001).

Based on the relative mobility on SDS-PAGE and the number of Cys residues, rice prolamins are grouped as follows: 10-/16-kDa prolamins, prolamin 13a-I/-II (Cys-rich 13-kDa prolamin), and prolamin 13b-I/-II (Cys-poor 13-kDa prolamin). To observe which prolamins are suppressed, group-specific primers were constructed from their conserved regions as listed in Supplementary Table S2. The result showed that the expression of genes encoding prolamin 13a-I and -II proteins are suppressed approximately 16 and 32 fold, respectively, in the GPGb-RNAi transgenic rice seeds compared to those of the wild-type cultivar (cv. *Ilmi*). The expression of genes encoding 16-kDa prolamin and prolamin 13b-II proteins showed to be also suppressed about twofold, whereas the expression of genes encoding prolamin 13b-I proteins are increased approximately 32 fold in the GPGb-RNAi transgenic rice seeds. Similarly, to observe the expression of *glutelin* genes clustered to GluA, GluB, GluC, and GluD groups, their specific primers were constructed as shown in Supplementary Table S2. **Figure 4A** showed that the expression of genes encoding globulin and GluA are suppressed about 8 and 128 fold, respectively, in the GPGb-RNAi transgenic rice seeds, whereas genes encoding GluB was found to be increased about twofold at transcript levels.

Both 2-DGE and Western blot analysis (**Figures 1C and 2**) showed that the ER-stress indicators, BiP1 and PDIL1-1, are increased in GPGb-RNAi transgenic rice seeds. As a next step and to further investigate which molecular events of the ER secretory system are activated in the GPGb-RNAi transgenic rice seeds, the expression of genes involved in ER-stress, including *OsBiP1, OsBiP2, OsSar1a, OsSar1b, OsSar1c, OsPDIL1-1, OsPDIL2*-3, and *CNX*, was examined (**Figure 4B**). The results showed that *OsBiP1* and *OsPDIL1-1* are commonly up-regulated about 1.5 and 2 fold, respectively, in the immature seeds of the GPGb-RNAi transgenic rice compared to those in the wild-type (cv. *Ilmi*) lines.

**Figure 3** indicated that the starch granules and starch contents are changed in the seed endosperms of the GPGb-RNAi transformants. Thus, we observed the expression of starch synthesis-related genes in the GPGb-RNAi transgenic rice seeds (**Figure 4C**), including granule-bound starch synthase (*OsGBSS*), ADP-glucose pyrophosphorylase small subunit (*OsAGPS1*), large subunit (*OsAGPL2, OsAGPL3*, and *OsAGPL4*), starch synthase (*OsSSII-3*) and starch branching enzyme (*OsSBE2* and *OsSBE3*). The expression of *OsAGPL3* and *OsSSII-3* were down-regulated (>1.5-fold) in the GPGb-RNAi transgenic rice seeds compared to wild-type.

## Discussion

### Morphological Changes in the Intracellular Organelles of Seed Endosperms of GPGb-RNAi Transgenic Rice

Various prolamin species accumulate in ER-derived PBs. Studies on PB formation have been well-characterized in maize. β- and γ-zeins corresponding to rice 10-kDa prolamins are aggregated into small PBs during endosperm development, then α- and δ-zeins corresponding to rice 13 and 16-kDa prolamins fill the interior of the PBs, expanding them (Wu and Messing, 2010). In rice, prolamins synthesized on the rough ER form type I PBs (PB-Is, spherical shape with a diameter 1–3 μm) in endosperm cells. Nagamine et al. (2011) reported that 10-kDa prolamins are in the electron-dense center of PB-Is, whereas Cys-poor prolamin 13b is in the periphery of PB-Is. Furthermore, Saito’s group showed that PB-Is consist of four regions, including a core region (10-kDa prolamin-rich), a inner layer (prolamin 13b-rich), a middle layer (prolamin 13a and 16-kDa prolamin-rich) and a outermost layer (prolamin 13b-rich) (Saito et al., 2012).

In SSP-silenced transgenic rice seeds, the reduction in the 13-kDa prolamin, including RM1 (pro13a.2 in prolamin 13a-I), RM2 (pro13b.2 in 13b-I), RM4 (pro13b.11/12/13 in 13b-II) and RM9 (pro13a.4 in 13a-II), resulted in the formation of many smaller PB-Is than in the wild-type rice seeds. The reduction in the 16-kDa prolamin showed little alternation in terms of the PB formation and the reduction in the 10-kDa prolamin resulted in the formation of smaller PB-Is without the change of PB-I’s spherical shape. Moreover, in the prolamin-RNAi transgenic rice seeds suppressing prolamin 13a and 13b family proteins, we observed that PB-Is are smaller than in the wild-type seeds and that the lamella within PB-Is disappeared (Kim et al., 2013). Similarly, PB-Is in the wild-type showed concentric layers of varying electron density according to the localized prolamin species (Supplementary Figures S4D,E). Prolamin 13a and the 16-kDa prolamin within the internal structure of rice PB-Is is accumulated on the heavily stained layer, and prolamin 13b is localized on the lightly stained layer between the outermost layer and the heavily stained layer (Saito et al., 2012). However, PB-Is in the endosperm cells of GPGb-RNAi transgenic rice showed abnormality, i.e., consisted of the lightly stained materials and that were smaller than those in the wild-type (Supplementary Figures S4L,M), resulting from the suppression of Cys-rich prolamin 13a and the enhancement of Cys-poor prolamin 13b-I.

Rice glutelin and globulin are synthesized on the ER and then transported to PSVs through the Golgi apparatus. Glutelins are stored in the inner region of PSVs, and globulin is distributed in the peripheral matrix surrounding the glutelins. Kawakatsu et al. (2010)proposed that globulin could play a role in constructing a frame for PSV formation and that the inside of the frame might be filled with glutelins, thus expanding the PVS. Indeed, in glutelin-less transgenic rice lines, including Glu-less, GluB-less and GluB⋅Glb-less, PVSs were relatively fewer and smaller compared to those in wild-type, suggesting that glutelins are responsible for the expansion of PSVs (Kawakatsu et al., 2010). In the Glb-RNAi transgenic rice with globulin expression blocked, the structure of the PSVs was cracked, and they were smaller and fewer in number than in the wild-type (Lee et al., 2015). In this study, the observation of GPGb-RNAi transgenic rice seeds (**Figures 1B,C, 2, and 4A**) showed that the GluA proteins and globulin are suppressed at protein and transcript levels but that GluB proteins are increased compared to those in the wild-type seeds, resulting in the formation of fewer and smaller PSVs (Supplementary Figure S4).

The decrease and increase in the PB-I proteins (prolamins) has been known to be compensated for through the corresponding increase and decrease of PSV proteins (glutelins and globulin). Consistently, the reduction of the 13-kDa prolamins resulted in the enhancement of glutelins and globulin in 13-kDa prolamin-less transgenic rice seeds (Kawakatsu et al., 2010; Kim et al., 2013). Conversely, the decrease in glutelins, including the GluA and GluB proteins, resulted in the increase of 13-kDa prolamins in Glu-less, GluB-less and GluB-/Glb-less transgenic rice seeds (Kawakatsu et al., 2010). However, in the GPGb-RNAi transgenic rice seeds simultaneously suppressing PB-I proteins (prolamin 13a) and PSV proteins (GluA and globulin), the GluB and prolamin 13b-I were observed to be increased at transcript and/or protein levels.

### Simultaneous Suppression of Glutelin, Prolamin, and Globulin Activates ER-Stress Responses in Rice Seed Endosperms

In the GPGb-RNAi transgenic rice seeds, which simultaneously suppress GluA, prolamin 13a and globulin, genes encoding ER chaperones, such as BiP and PDI (but not CNX), were induced at the transcript and protein levels (**Figures 1C, 2, and 4B**). SSPs synthesized during seed maturation are deposited to the PBs from ER lumen via a process mediated by ER chaperones. However, the excessive deposition of SSPs or some recombinant proteins on the ER lumen induces the aberrant expression of ER chaperones, resulting in ER-stress responses accompanied by abnormal intracellular structures and floury phenotypes (Oono et al., 2010).

Previous studies on loss of SSP function showed that the reduction in the SSPs is compensated for by other SSPs. For example, in PSV protein-deficient transgenic rice seeds, prolamin 13b proteins (Cys-poor 13-kDa prolamin) were preferentially increased, resulting in ER-stress responses. In 13-kDa Pro-less transgenic rice seeds, all of the Cys-poor prolamins were likely suppressed, inducing a similar result observed in PSV protein-deficient lines (Kawakatsu et al., 2010). Furthermore, maize *opaque2* (a basic leucine zipper transcription factor) mutant showed a floury kernel phenotype with an increase in the lysine content and a reduction in the 22-kDa α-zein similar to rice 10-kDa prolamin (Schmidt et al., 1990; Segal et al., 2003). RNAi-mediated suppression of the α-zein resulted in a floury kernel phenotype (Wu and Messing, 2010). Based on the sum of these results, Kawakstsu and co-workers proposed that the deficiency of Cys-rich prolamins or Cys-poor prolamins breaks the steady ratio of Cys-poor to Cys-rich prolamins, disrupting the proper aggregation and folding of prolamins and resulting in ER-stress responses such as BiP induction (Kawakatsu et al., 2010). Indeed, BiP-overexpressing rice seeds suppress SSPs and starch contents, displaying an opaque phenotype with floury and shrunken features (Takaiwa et al., 2009; Yasuda et al., 2009; Wakasa et al., 2011). In this study, Western blotting, qRT-PCR and TEM analysis (**Figures 1C and 4A**; Supplementary Figure S4) showed the suppression of prolamin 13a (Cys-rich 13-kDa prolamin), the increase of prolamin 13b-I (Cys-poor 13-kDa prolamin) and the morphological change of PB-Is in GPGb-RNAi transgenic rice seeds, indicating the change of the steady ratio of Cys-rich to Cys-poor prolamins. This change might induce the ER-stress responses, resulting in the floury feature of rice seeds.

The over-expression of PDI, one of main ER chaperones activated by ER-stress, leads to a seed phenotype similar to that of the wild-type (Yasuda et al., 2009). However, in mutants (*esp2*) with a loss of function of PDIL1-1, proglutelins abnormally accumulate in the ER lumen and interact with prolamin polypeptides, forming new PBs containing both proglutelin and prolamin (Takemoto et al., 2002; Satoh-Cruz et al., 2010). The results indicated the involvement of PDIL1-1 in the processing of proglutelins to mature glutelin forms. In addition, OsSar1, a small GPTase protein, is one component of coat protein complex II (COPII), which has been implicated in the transport system of proglutelin and globulin from ER to Golgi apparatus in rice endosperm (Barlowe, 2002). Simultaneous knockdown of *OsSar1a*/*b*/*c* resulted in floury and shrunken seeds with increased levels of proglutelin, BiP1 and PDI 2-3 and decreased the levels of mature glutelin subunits (Tian et al., 2013). Herein, the simultaneous suppression of GluA, prolamin 13a and globulin induced increases in the OsBiP1 and OsPDIL1-1 but not OsBiP2, OsCNX, and OsSar1a/b/c. These results point toward a relation between increase in OsBiP1 and OsPDIL1-1 and the observed intracellular structural changes and floury phenotypes in GPGb-RNAi transgenic rice seeds.

### Seed Germination of GPGb-RNAi Transgenic Rice is Delayed

ER is an essential cellular organelle, regulating many proteins involved in protein folding, trafficking and energy metabolism. Abnormal conditions of ER such as unfold protein responses regulate the transcription of genes related to glucose synthesis or breakdown pathways (Hotamisligil, 2010). The suppression or overexpression of BiP1 in rice endosperm led to ER stress, resulting in the change of expression patterns of starch synthesis-related genes and floury seeds (Wakasa et al., 2011). Moreover, proteomic analysis in ER-stressed rice seeds revealed changes in expression of carbohydrate metabolism-related proteins (Qian et al., 2015). Thus, the enhanced expression of ER chaperones such as OsBiP1 and OsPDIL1-1 in GPGb-RNAi transgenic rice seeds indicates that ER is an abnormal state, probably influencing carbohydrate metabolism. Indeed, the transcription levels of starch synthesis-related genes such as *OsGBSS, OsAGPL3, OsAGPL4, OsSSII-3*, and *OsSBE3* were found to be significantly lower in GPGb-RNAi transgenic rice seeds than those of the wild-type cultivar, most probably affecting the starch quality. SEM images and starch content analysis (**Figure 3**) revealed that the starch granules are loosely packed and the starch contents are decreased in the GPGb-RNAi transgenic rice seeds, resulting in floury seeds.

**FIGURE 5:**
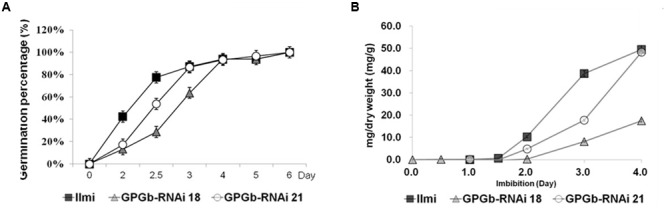
**Kinetics of germination and accumulation of reducing sugars in wild-type and GPGb-RNAi transgenic rice seeds. (A)** Germination percentage of seeds of wild-type and GPGb-RNAi 18 and 21 transgenic lines are calculated as the mean of three biological replicates, on the basis of the total number of germinated seeds on the last day. Seeds were sterilized in 2% hypochlorite solution and soaked in distilled water at 25°C for 6 days. **(B)** The reducing sugar contents (mg/dry weight) are measured in seeds germinated on days 0, 0.5, 1, 1.5, 2, 3, and 4 and represented as the mean of three biological replicates. Error bars indicate the standard deviation (SD).

Furthermore, to investigate the features of the GPGb-RNAi transgenic rice seeds, we performed their germination test. RNAi-based individual suppression of glutelins (Glu-RNAi), and globulin (Glb-RNAi) showed that their germination rates are similar to those of the wild-type seeds. However, the germination rate of the GPGb-RNAi transgenic rice seeds was remarkably delayed compared to that of the wild-type seeds although the total number of germinated seeds is similar to the wild-type (**Figure 5**; Supplementary Figure S4), indicating that the seed germination condition of the GPGb-RNAi transgenic rice is more inferior compared to those of the Glu-RNAi and Glb-RNAi transgenic rice and wild-type. Zuber et al. (2013) reported that the germination of sulfur-deficient legume seeds is delayed compared to wild-type seeds, suggesting that the sulfur deficiency during germination weakly activates metabolite enzymes such as chlorophyll synthesis and glycolysis, resulting in a delay in germination. GPGb-RNAi transgenic rice seeds were found to suppress prolamin 13a (Cys-rich 13-kDa prolamin) but enhanced prolamin 13b-I (Cys-poor 13-kDa prolamin) at the transcript and/or protein levels (**Figures 1C and 4A**). These changes might cause a decrease in the sulfur source within the SSPs, resulting in the germination delay. However, with a possibility of other causes for the delayed germination in the GPGb-RNAi transgenic seeds remaining, there is a need for additional experimental evidences in the future.

## Author Contributions

KC, H-JL, and Y-MJ designed and carried out the experiments. KC and H-JL analyzed the results and wrote the manuscript. J-YL and Y-MK designed the research, contributed scientific advice, and corrected the manuscript. S-HL and RR contributed scientific advice, critical reading and revision and editing of the manuscript. All authors have read and approved finally to submit the manuscript.

## Conflict of Interest Statement

The authors declare that the research was conducted in the absence of any commercial or financial relationships that could be construed as a potential conflict of interest.
