# Supplementary material for: RNA Interference-Mediated Simultaneous Suppression of Seed Storage Proteins in Rice Grains
Source: Front Plant Sci. 2016 Oct 31;7:1624. doi: 10.3389/fpls.2016.01624 (PMC5087109; doi:10.3389/fpls.2016.01624)
Supplement: Supplementary file 1 [file Presentation_1.PDF]

Yellow: cDNA fragment of glutelin A-2 (Os10g0400200, 229 bp)

```
CAGTTTGCTTGTTCCTCTTGTGCGATGGCTCCCTAGCCCAGCAGCTATTAGGCCAGAGCACTAGTCAATG  70

GCAGAGTTCTCGTCGTGGAAGTCCGAGAGGATGTAGATTTGATAGGTTGCAAGCATTTGAGCCAATTCGG  140

AGTGTGAGGTCTCAAGCTGGCACAACTGAGTTCTTCGATGTCTCTAATGAGTTGTTTCAATGTACCGGAG  210

TATCTGTTGTCCGCCGAGTTTTGCTCTCCTTGCTATTGTTGCATGCAACGCTTCTGCACGGTTTGATGCT  280

CTTAGTCAAAGTTATAGACAATATCAACTACAATCGCATCTCCAGCTACAGCAACAAGTGCTCAGCCCAT  350

GCAGTGAGTTCGTAAGGCAACAGCATAGCATAGTGGCAACCCCCTTCTGGCAACCAGCTACGTTTCAATT  420
```

Green: cDNA fragment of prolamin 13a.2 (Os07g0206500, 505 bp)

```
GATAAACAACCAAGTCATGCAGCAACAGTGTTGCCAACAGCTCAGGCTGGTAGCGCAACAATCTCACTAC  490

CAGGCCATTAGTAGCGTTCAGGCGATTGTGCAGCAACTACAGCTGCAGCAGGTCGGTGTTGTCTACTTTG  560

ATCAGACTCAAGCTCAAGCTCAAGCTTTGCTGGCCTTAAACTTGCCATCCATATGTGGTATCTATCCTAA  630

CTACTACATTGCTCCGAGGAGCATTCCCACCGTTGGTGTGTCTGGTACTGAATTGTAATAGTATAATGGT  700

TCAAATGTTAAAAATAAAGTCATGCATCATCATGGAGAGGTTCCAGCCGATGTTCCGCCGCCCGGGCGCG  770

CTCGGCCTGCGGATGCAGTGCTGCCAGCAGCTGCAGGACGTGAGCCGCGAGTGCCGCTGCGCCGCCATCC  840
```

Gray: cDNA fragment of α-globulin (Os05g0499100, 305 bp)

```
GCCGGATGGTGAGGAGCTACGAGGAGAGCATGCCGATGCCCCTGGAGCAAGGCTGGTCGTCGTCGTCGTC  910

GGAGTACTACGGCGGCGAGGGGTCGTCGTCGGAGCAGGGGTACTACGGCGAGGGGTCGTCGGAGGAGGGC  980

TACTACGGCGAGCAGCAGCAGCAGCCGGGGATGACCCGCGTGAGGCTGACCAGGGCGAG            1039
```

Figure S1. Nucleotide sequence of the linked glutelin-prolamin-globulin cDNA fragments. The linked cDNA was used to construct binary vector to generate GPGb-RNAi transgenic rice plants suppressing glutelin, prolamin and globulin genes.

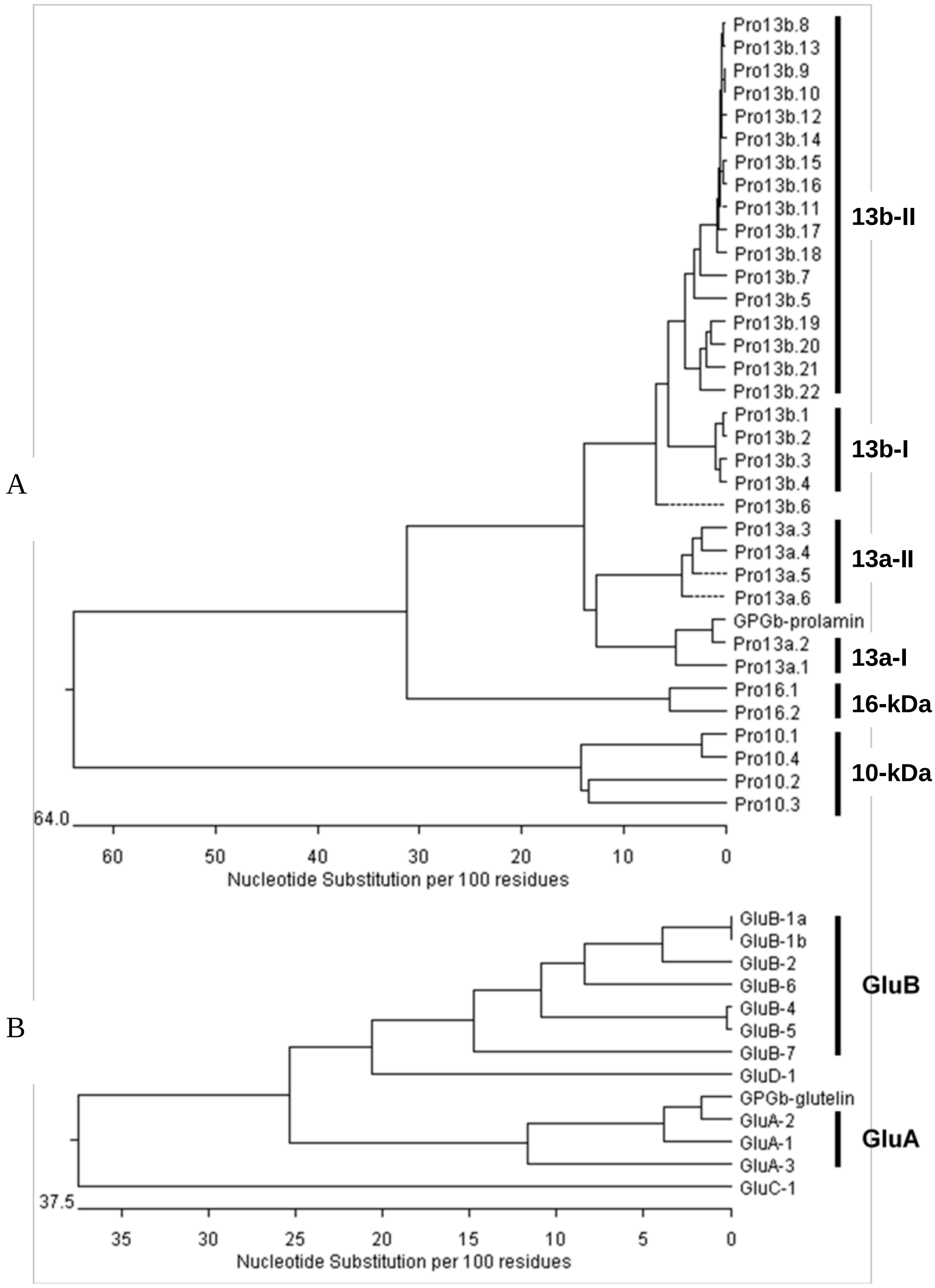

Figure S2. Phylogenetic tree analysis of the conserved regions within prolamin 13a-I (A) and glutelin A (B) family. The conserved nucleotide sequences were produced from *Pro13a.2* (Os06g0206500; GPGb-prolamin: 505 bp) and *GluA-2* (Os10g0400200; GPGb-glutelin: 229bp), respectively. They were aligned with nucleotide sequences of rice 34 prolamin genes (A, Saito et al., 2012) and 12 glutelin genes (B, Kawakatsu et al., 2008) using ClustalW in MegAlign software (v. 8.1.4).

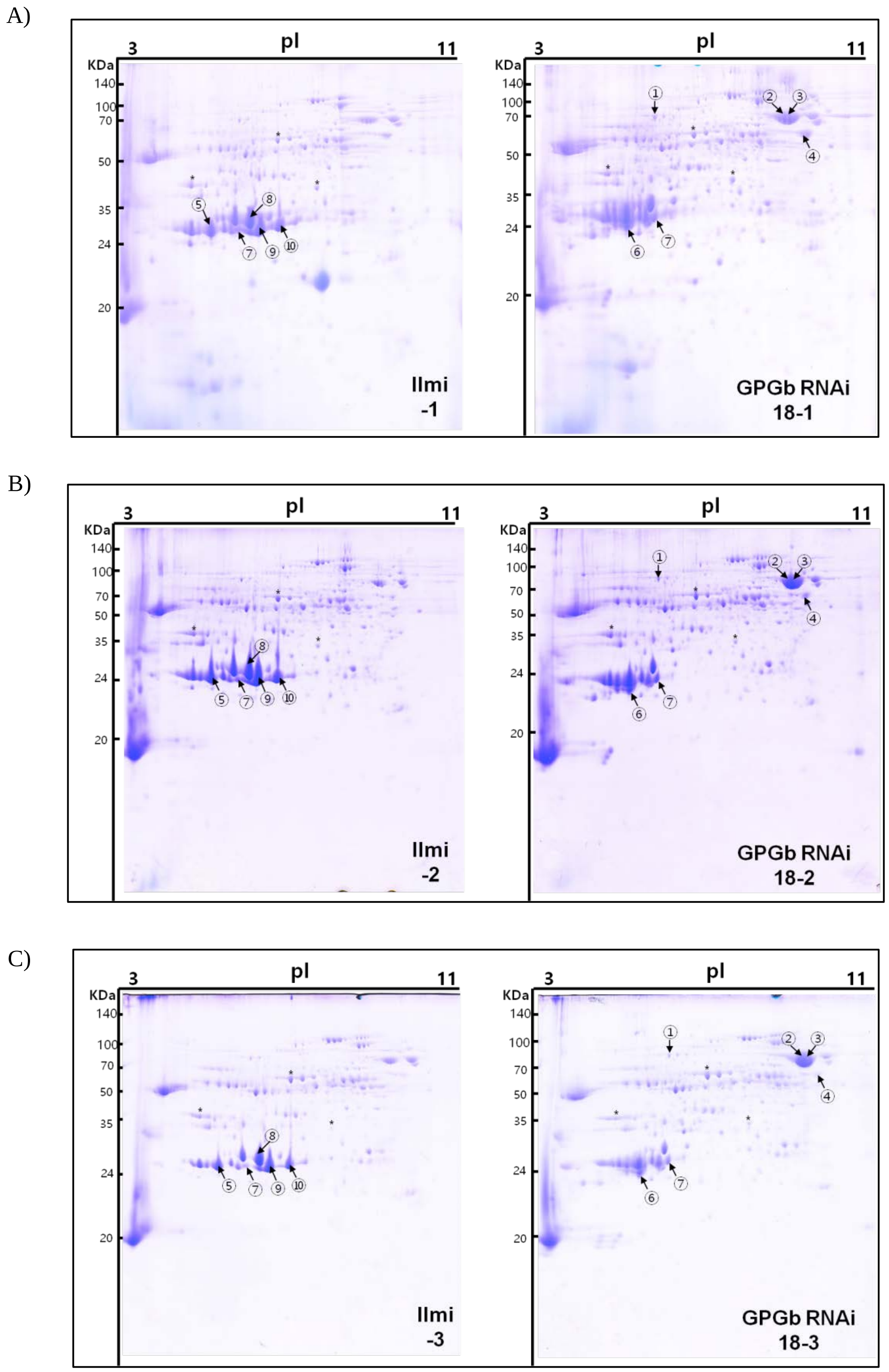

Figure S3. Two-dimensional gel electrophoresis (2-DGE). To ensure reproducibility, total seed proteins of wild-type (cv. *Ilmi*) and GPGb-RNAi transformants were extracted from three independent experiments (A, B and C) and then analyzed by 2-DGE with 50 (A and C) and 100 µg (B) of SSPs. Ten interesting spots were selected as differentially expressed protein spots between the wild-type and GPGb-RNAi transgenic rice seeds. Asterisks denoted the landmarks to indicate same spot positions across all 2-D gels.

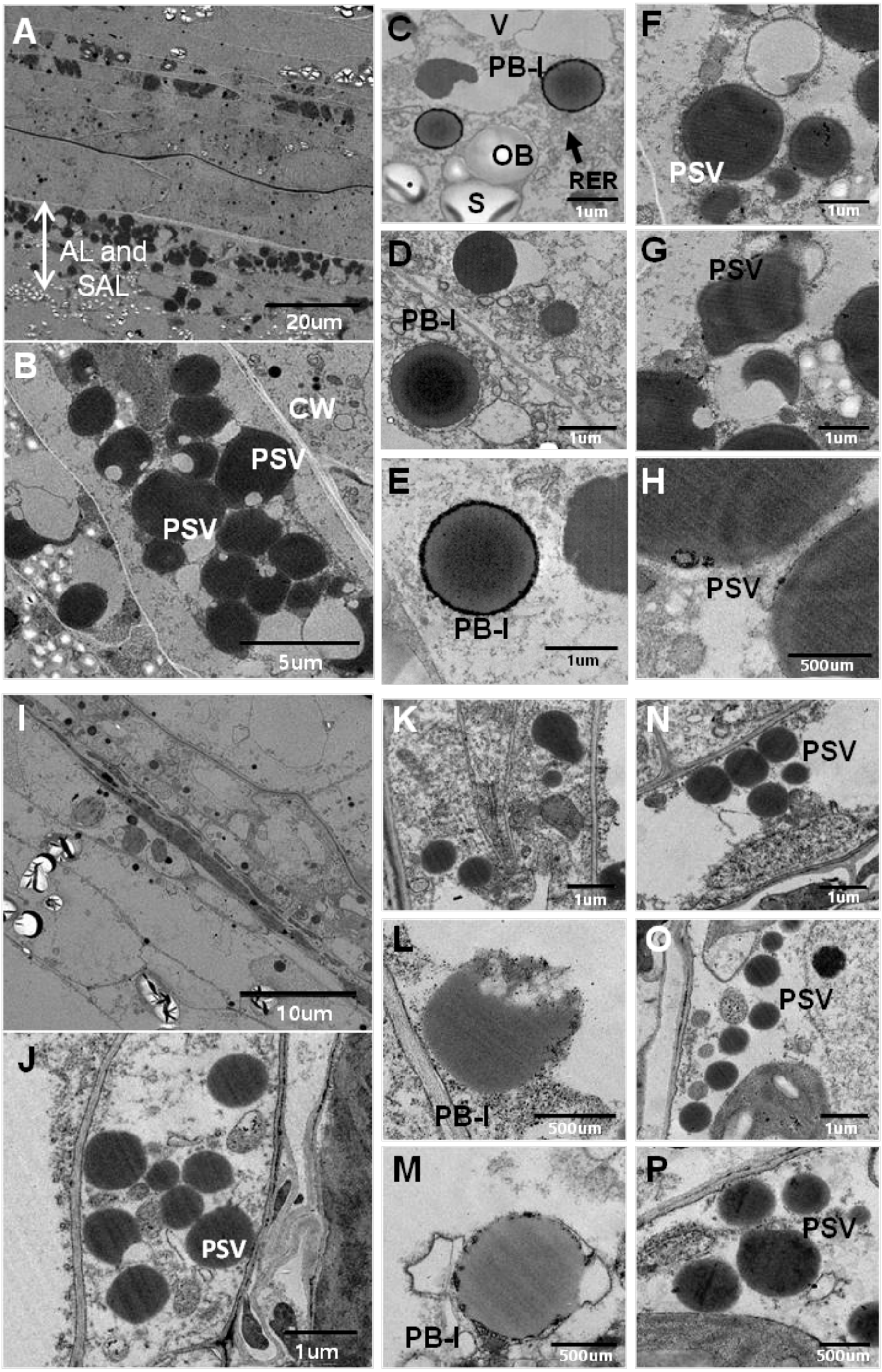

Figure S4. Phenotypic characterization of the seed storage organelles. TEM was used to observe protein bodies in developing seeds of the wild-type (A–H) and GPGb-RNAi transformants (I–P). Scale bar is indicated on each panel. AL, aleurone layer; SAL, subaleurone layer; CW, cell wall; V, vacuole; S, starch grain; OB, oil body; PB-I, protein body-I; PSV, protein storage vacuole; and RER, rough endoplasmic reticulum.

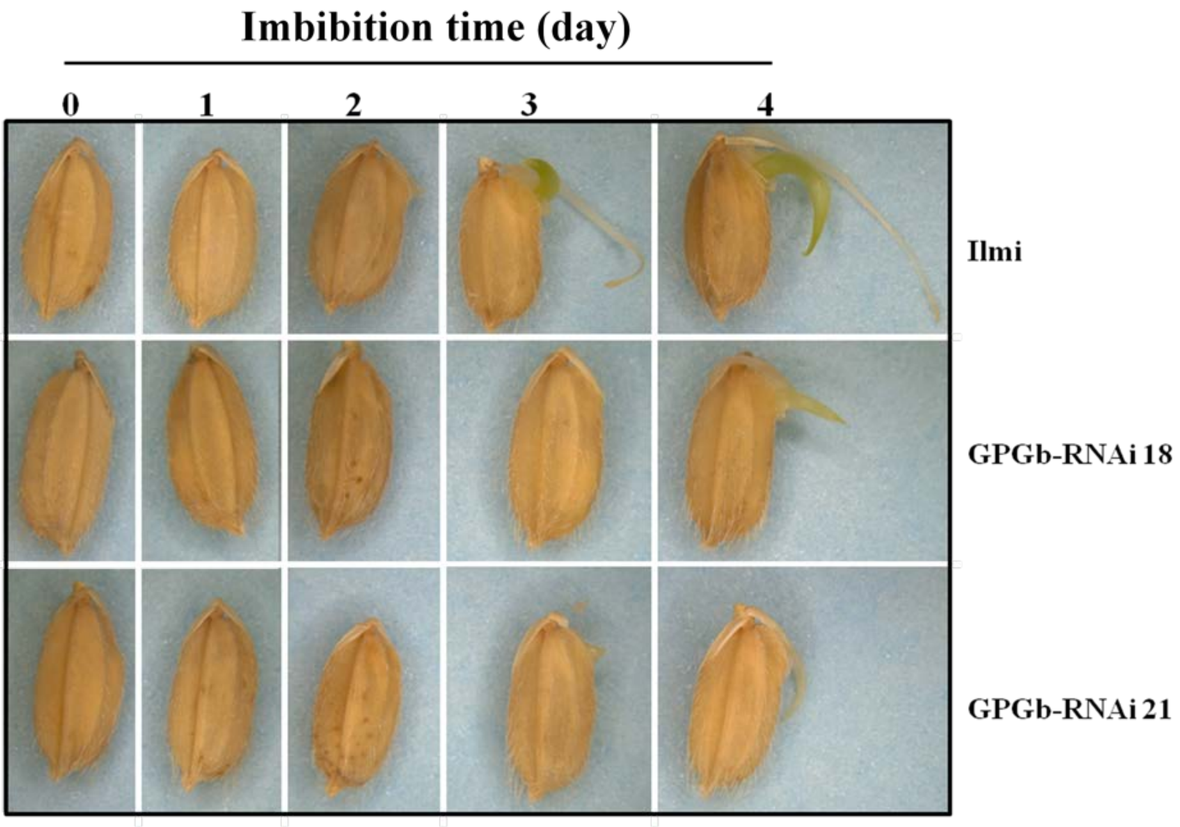

Figure S5. Germination patterns of GPGb-RNAi line seeds. Seeds harvested from GPGb-RNAi transgenic rice were sterilized with 70% ethanol for 5 min and washed with distilled water for 10 min three times. Seeds were then sterilized with 2% hypochlorite solution for 30 min and rinsed with distilled water for 10 min five times. The sterilized seeds were soaked in distilled water and incubated at 25˚C for 4 days in clean room (light/16 h and darkness/8 h).

Supplementary Table 1. MS/MS peptide sequences and identification of the selected protein spots.

Spot #	Gene ID	Name	Score	Cov. (%)	pI/ MW	Amino Acid sequence*
Spot 1	Os03g02 71200	Chloroplastic membrane protein precursor	17.19	17.40	8.51 / 87.69	MALTSQSLFFSPLAAGPSRRVRGRGRSTSVSAAASASSHNSQPHGHPQQPLAVASSSSKSESKGSKTFALASAITAAASGAFLLASSGGGFGGGAGGPLGGGGGGWGAGGGGGGGGGGGGGGFWSRIFSGGAAHADEKSSGDWDPHGLPANINVPMTKLSGLKRYKISELKFFDRAAGGGGAFTGPEDSFFEMVTLQPGGVYTKSQLLKELETLVSCGMFERVDLEGKAKPDGTLGLTVSFVESVWSAAKQFKCINVGLMSQSGQVDFDQDMTEREKMDYLRKQERDYQQRVRGAKPCILPDNVRGEVLGMMKKQEKVSARLLQRIRDHVQKWYHNEGFVCAQVVNFGNLNTSEVVCEVVEGDITKVEYQFQDK**LGNFVEGNTQIPIIDR**ELPQQLRPGHIFNIGAGKQALKNINSLALFSNIEVNPRPDETKEGGIVVEIKLKELEPK**SAEVSTEWSIVPGREGRPTLASIQPGGTVSFEHR**NIYGLNRSIVGSVTSSNLLNPQDDLSFKLEYVHPYLDGVDDRNKNRTFKTSCFNTRKLSPVFVAGPNMDEAPPVWVDRVGFKANITESFTRQSKFTYGLVVEEITTRDETNSICTHGSRAMPSGGLSMDGPPTTLSGTGIDRMAFLQANITRDNTEFVNGAVIGDRCIFQLDQGLGIGSKNPFFNRHQLTLTKFVNLNKQEKGAGKPLPAVLVLHGHYAGCVGDLPSYDAFTLGGPYSVRGYGMGELGASR**NVLEVASELR**IPVR**NTYVYGFVEHGTDLGSSK**DVKGNPTEFFRRVGHGSSYGLGVKLGLVR**GEYIVDHNAGTGTVFFR**FGERF
Spot 2	Os02g01 15900	Putative chaperone BiP	633.97	51.43	5.09 / 73.39	MDRVRGCAFLLGVLLAGSLFAFSVAKEETKKLGTVIGIDLGTTYSCVGVYK**NGHVEIIANDQGNRITPSWVAFTDSERLIGEAAKNQAAVNPER**TIFDVKRLIGR**KFEDKEVQR**DMKLVPYK**IVNKDGKPYIQVK**IKDGENKVFSPEEVSAMILGKMK**ETAEAYLGKKINDAVVTVPAYFNDAQR**QATK**DAGVIAGLNVARIINEPTAAAIAYGLDKK**GGEKNILVFDLGGGTFDVSILTIDNGVFEVLATNGDTHLGGEDFDQRIMEYFIKLIKKK**YSKDISKDNR**ALGKLRREAERAK**RALSNQHQVRVEIESLFDGTDFSEPLTRARFEELNNDLFRK**TMGPVKKAMDDAGLEK**SQIHEIVLVGGSTR**IPKVQQLLR**DYFEGKEPNKGVNPDEAVAYGAAVQGSILSGEGGDETK**DILLLDVAPLTLGIETVGGVMTKLIPR**NTVIPTKKSQVFTTYQDQQTTVSIQVFEGER**SMTKDCR**LLGKFDLSGIPAAPR**GTPQIEVTFEVDANGILNVKAEDKGTGKSEK**ITITNEKGRLSQEEIDR**MVR**EAEEFAEEDKKVK**ERIDARNQLETYVYNMKNTVGDKDKLADKLESEEKEKVEEALK**EALEWLDENQTAEKEEYEEK**LKEVEAVCNPIISAVYQR**TGGAPGGGADGEGGVDDEHDEL**
Spot 3	Os02g01 15900	Putative chaperone BiP	564.96	47.52	5.09 / 73.39	MDRVRGCAFLLGVLLAGSLFAFSVAKEETKKLGTVIGIDLGTTYSCVGVYK**NGHVEIIANDQGNRITPSWVAFTDSER**LIGEAAK**NQAAVNPER**TIFDVKRLIGR**KFEDKEVQR**DMKLVPYK**IVNKDGKPYIQVK**IKDGENKVFSPEEVSAMILGKMK**ETAEAYLGKKINDAVVTVPAYFNDAQR**QATK**DAGVIAGLNVARIINEPTAAAIAYGLDKK**GGEKNILVFDLGGGTFDVSILTIDNGVFEVLATNGDTHLGGEDFDQRIMEYFIKLIKKK**YSKDISKDNR**ALGKLRREAERAK**RALSNQHQVRVEIESLFDGTDFSEPLTRARFEELNNDLFRK**TMGPVKKAMDDAGLEK**SQIHEIVLVGGSTR**IPKVQQLLR**DYFEGKEPNKGVNPDEAVAYGAAVQGSILSGEGGDETK**DILLLDVAPLTLGIETVGGVMTKLIPRNTVIPTKKSQVFTTYQDQQTTVSIQVFEGERSMTKDCR**LLGKFDLSGIPAAPR**GTPQIEVTFEVDANGILNVKAEDKGTGKSEK**ITITNEKGRLSQEEIDR**MVR**EAEEFAEEDKKVK**ERIDARNQLETYVYNMKNTVGDKDK**LADKLESEEKEK**VEEALK**EALEWLDENQTAEKEEYEEK**LKEVEAVCNPIISAVYQR**TGGAPGGGADGEGGVDDEHDEL**
Spot 4	Os11g01 99200	PDIL 1-1	70.82	24.80	5.01 / 56.85	MAISKAWISLLLALAVVLSAPAARAEEAAAAEEGGDAAAEAVLTLDADGFDEAVAKHPFMVVEFYAPWCGHCK**KLAPEYEK**AAQELSK**HDPPIVLAKVDANDEKNKPLATK**YEIQGFPTLKIFRNQGKNIQEYKGPR**EAEGIVEYLK**KQVGPASK**EIKSPEDATNLIDDK**KIYIVGIFSELSGTEYTNFIEVAEKLRSDYDFGHTLHANHLPRGDAAVERPLVRLFKPFDELVVDSKDFDVTALEK**FIDASSTPK**VVTFDKNPDNHPYLLKFFQSSAAKAMLFLNFSTGPFESFKSVYYGAAEEFKDKEIKFLIGDIEASQGAFQYFGLREDQVPLIIIQDGESKKFLKAHVEPDQIVSWLKEYFDGKLSPFR**KSEPIPEVNDEPVK**VVVADNVHDFVFKSGKNVLVEFYAPWCGHCKK**LAPILDEAATTLKSDKDVVIAK**MDATANDVPSEFDVQGYPTLYFVTPSGKMVPYESGR**TADEIVDFIK**KNKETAGQAK**EKAESAPAEPLKDEL**
Spot 5	Os02g02 49800	Glutelin type-B 1	274.97	23.27	9.26 / 56.55	MASSVFSRFSIYFCVLLLCHGSMAQLFNPSTNPWHSPRQGSFRECR**FDRLQAFEPLRKVRSEAGVTEYFDEK**NELFQCTGTFVIR**RVIQPQGLLVPRYTNIPGVVYIIQGR**GSMGLTFPGCPATYQQQFQQFSSQGQSQSQKFRDEHQKIHQFRQGDIVALPAGVAHWFYNDGDAPIVAVYVYDVNNNANQLEPR**QKEFLLAGNNNR**AQQQQVYGSSIEQHSGQNIFSGFGVEMLSEALGINAVAAK**RLQSQNDQRGEIIHVKNGLQLLKPTLTQQQEQAQAQDQYQQVQYSER**QQTSSRWNGLEENFCTIKVRVNIENPSRADSYNPRAGRITSVNSQKFPILNLIQMSATRVNLYQNAILSPFWNVNAHSLVYMIQGRSRVQVVSNFGKTVFDGVLRPGQLLIIPQHYAVLKKAEREGCQYIAIKTNANAFVSHLAGKNSVFRALPVDVVANAYRISREQARSLKNNRGEEHGAFTPRFQQQYYPGLSNESESETSE
Spot 6	Os02g02 68100	Glutelin type-B 5	254.29	44.87	9.00 / 56.81	MATIAFSRLSIYFCVLLLCHGSMAQLFGPNVNPWHNPRQGGFRECR**FDRLQAFEPLRRVRSEAGVTEYFDEK**NEQFQCTGTFVIR**RVIEPQGLLVPRYSNTPGMVYIIQGRGSMGLTFPGCPATYQQQFQQFLPEGQSQSQK**FRDEHQKIHQFRQGDIVALPAGVAHWFYNEGDAPVVALYVFDLNNNANQLEPR**QKEFLLAGNNNR**EQQMYGR**SIEQHSGQNIFSGFNNELLSEALGVNALVAKRLQGQNDQRGEIIR**VKNGLKLLRPAFAQQQEQAQQQEQAQAQYQVQYSEEQQPSTRCNGLDENFCTIKARLNIENPSHADTYNPRAGRITRLNSQKFPILNLVQLSATRVNLYQNAILSPFWNVNAHSLVYIVQGHARVQVVSNLGKTVFNGVLRPGQLLIIPQHYVVLKKAEHEGCQYISFKTNANSMVSHLAGKNSIFRAMPVDVIANAYRISREQARSLKNNRGEELGAFTPRYQQQTYLGFSNESENEASE

Supplementary Table 1. MS/MS peptide sequences and identification of the selected protein spots (continued).

Spot #	Gene ID	Name	Score	Cov. (%)	pI/ MW	Amino Acid sequence*
Spot 7	Os02g02 49600	Glutelin type-B 2	100.03	12.79	9.11 / 56.05	MATTIFSRFSIYFCAMLLCQGSMAQLFNPSTNPWHSPRQGSFRECR**FDRLQAFEPLRK**VR**SEAGVTEYFDEK**NELFQCTGTFVIR**RVIQPQGLLVPR**YSNTPGLVYIIQGRGSMGLTFPGCPATYQQQFQQFSSQGQSQSQKFRDEHQKIHQFRQGDVVALPAGVAHWFYNDGDASVVAIYVYDINNSANQLEPR**QKEFLLAGNNNR**VQQVYGSSIEQHSSQNIFNGFGTELLSEALGINTVAAKRLQSQNDQRGEIVHVKNGLQLLKPTLTQQQEQAQAQYQEVQYSEQQQTSSRWNGLEENFCTIKARVNIENPSRADSYNPRAGRISSVNSQKFPILNLIQMSATRVNLYQNAILSPFWNVNAHSLVYMIQGQSRVQVVSNFGKTVFDGVLRPGQLLIIPQHYAVLKKAEREGCQYIAIKTNANAFVSHLAGKNSVFRALPVDVVANAYRISREQARSIKNNRGEEHGAFTPRFQQQYYPGFSNESESETSE
Spot 8	Os10g04 00200	Glutelin type-A 2	174.63	27.91	8.93 / 56.31	MASINRPIVFFTVCLFLLCDGSLAQQLLGQSTSQWQSSRRGSPRGCR**FDRLQAFEPIR**SVRSQAGTTEFFDVSNELFQCTGVSVVRRVIEPR**GLLLPHYTNGASLVYIIQGR**GITGPTFPGCPETYQQQFQQSGQAQLTESQSQSHK**FKDEHQKIHR**FRQGDVIALPAGVAHWCYNDGEVPVVAIYVTDINNGANQLDPR**QRDFLLAGNKRNPQAYR**R**EVEEWSQNIFSGFSTELLSEAFGISNQVAR**QLQCQNDQRGEIVRVER**GLSLLQPYASLQEQEQGQMQSREHYQEGGYQQSQYGSGCPNGLDETFC**TMRVRQNIDNPNRADTYNPRAGRVTNLNSQNFPILNLVQMSAVKVNLYQNALLSPFWNINAHSIVYITQGRAQVQVVNNNGKTVFNGELRRGQLLIVPQHYVVVKKAQREGCAYIAFKTNPNSMVSHIAGKSSIFRALPTDVLANAYRISREEAQRLKHNRGDEFGAFTPLQYKSYQDVYNVAESS
Spot 9	Os01g07 62500	Glutelin type-A 1	671.24	26.85	9.09 / 56.25	MASINRPIVFFTVCLFLLCNGSLAQQLLGQSTSQWQSSRRGSPRECR**FDRLQAFEPIR**SVRSQAGTTEFFDVSNEQFQCTGVSVVRRVIEPR**GLLLPHYTNGASLVYIIQGR**GITGPTFPGCPESYQQQFQQSGQAQLTESQSQSQKFKDEHQKIHRFRQGDVIALPAGVAHWCYNDGEVPVVAIYVTDLNNGANQLDPR**QRDFLLAGNKRNPQAYR**REVEER**SQNIFSGFSTELLSEALGVSSQVAR**QLQCQNDQR**GEIVRVEHGLSLLQPYASLQEQEQGQVQSRERYQEGQYQQSQYGSGCSNGLDETFCTLRVR**QNIDNPNRADTYNPRAGRVTNLNTQNFPILSLVQMSAVKVNLYQNALLSPFWNINAHSVVYITQGRARVQVVNNNGKTVFNGELRRGQLLIIPQHYAVVKKAQREGCAYIAFKTNPNSMVSHIAGKSSIFRALPNDVLANAYRISREEAQRLKHNRGDEFGAFTPIQYKSYQDVYNAAESS
Spot 10	Os03g04 27300	Glutelin type-A 3	463.17	25.17	8.81 / 56.02	MATIKFPIVFSVVCLFLLCNGSLAQLLSQSTSQWQSSRRGSPRECR**FDRLQAFEPIR**TVRSQAGTTEFFDVSNELFQCTGVFVVRRVIEPR**GLLLPHYSNGATLVYVIQGR**GITGPTFPGCPETYQQQFQQSEQDQQLEGQSQSHKFRDEHQKIHRFQQGDVVALPAGVAHWCYNDGDAPIVAIYVTDIYNSANQLDPR**HRDFFLAGNNKIGQQLYR**YEARDNSK**NVFGGFSVELLSEALGISSGVAR**QLQCQNDQRGEIVR**VEHGLSLLQPYASLQEQQQEQVQSRDYGQTQYQQK**QLQGSCSNGLDETFCTMRVRQNIDNPNLADTYNPRAGRITYLNGQKFPILNLVQMSAVKVNLYQNALLSPFWNINAHSVVYITQGRARVQVVNNNGKTVFDGELRRGQLLIIPQHHVVIKKAQREGCSYIALKTNPDSMVSHMAGKNSIFRALPDDVVANAYRISREEARRLKHNRGDELGVFTPSHAYKSYQDISVSA
Spot 11	Os03g04 27300	Glutelin type-A 3	29.18	11.91	8.81 / 56.02	MATIKFPIVFSVVCLFLLCNGSLAQLLSQSTSQWQSSRRGSPRECRFDR**LQAFEPIR**TVRSQAGTTEFFDVSNELFQCTGVFVVRRVIEPR**GLLLPHYSNGATLVYVIQGR**GITGPTFPGCPETYQQQFQQSEQDQQLEGQSQSHKFRDEHQKIHRFQQGDVVALPAGVAHWCYNDGDAPIVAIYVTDIYNSANQLDPRHRDFFLAGNNKIGQQLYRYEARDNSKNVFGGFSVELLSEALGISSGVARQLQCQNDQRGEIVR**VEHGLSLLQPYASLQEQQQEQVQSR**DYGQTQYQQKQLQGSCSNGLDETFCTMRVRQNIDNPNLADTYNPRAGRITYLNGQKFPILNLVQMSAVKVNLYQNALLSPFWNINAHSVVYITQGRARVQVVNNNGKTVFDGELRRGQLLIIPQHHVVIKKAQREGCSYIALKTNPDSMVSHMAGKNSIFRALPDDVVANAYRISREEARRLKHNRGDELGVFTPSHAYKSYQDISVSA

Red letter represents the peptide sequences identified from MS spectra

Score is the sum of Xcorr value of the peptides detected using SEQUEST search

Supplementary Table S2. List of primers.

Symbol	Name	Forward primer	Reverse primer	Target genes (Locus ID)	Product size
**Seed Storage Protein**					
Glb	Gluobulin	AGTCGGAGATGAGGTTCAGG	GAACATCGGCTGGAACCTC	Glb (Os05g0499100)	122
10-kDa	10-kDa prolamin	TTATTTGTGCTGGACTCGGG	GAGAGTTGGAAGTTGACAGGG	[a]pro10.1 (Os03g0766100), [a]pro10.4 (Os11g0535525)	97
13a-I	Prolamin 13a-I	CAACTACAGTCGCATCTCCTAC	GGGTTGCCACTATGCTATACTG	[a]pro13a.1 (Os07g0206400), [a]pro13a.2 (Os07g0206500)	88
13a-II	Prolamin 13a-II	TCACCCGTGTTTCAACTGAG	CACAATAGCCTGAACACTGC	[a]pro13a.4 (Os12g0269200), [a]pro13a.5 (Os12g0269600), [a]pro13a.6 (Os12g0269700)	117
13b-I	Prolamin 13b-I	TCAAGCTCAAGCACTGTTGG	AGTACAAGACACCGCCAAGG	[a]pro13b.1 (Os07g0219250), [a]pro13b.2 (Os07g0219300), [a]pro13b.3 (Os07g0219400), [a]pro13b.4 (Os07g0220000)	107
13b-II	Prolamin 13b-II	GCTCTGTTGGCTTTTAACGTG	ACTCATTACAAGACACCGCC	[a]pro13b.9 (Os05g0328901) ,[a]pro13b.10 (Os05g0329001), [a]pro13b.19 (Os05g0331366), [a]pro13b.20 (Os05g0331532), [a]pro13b.21 (Os05g0331800), [a]pro13b.22 (Os05g0332000)	101
16-kDa	16-kDa prolamin	CTCAATTTGCCCTCCATGTG	AGAACCGCAATGACCAGTAG	[a]pro16.1 (Os06g0507100), [a]pro16.2 (Os06g0507200)	81
GluA	Glutelin A	AATGATGGTGAAGTGCCGGT	TCACGCCTGTATGCTTGAGG	[b]GluA-1 (Os01g0762500), [b]GluA-2 (Os10g0400200)	131
GluB	Glutelin B	ATTGAGCAACACTCTGGGCA	TGGCTCTGTAGCCTCTTTGC	[b]GluB-1a (Os02g0249800), [b]GluB-1b (Os02g0249900), [b]GluB-4 (Os02g068300), [b]GluB-5 (Os02g068100)	104
GluC	Glutelin C-1	CACAAGGGCCAATAGCCAGA	GGTCACGTACATCACCGTGT	[b]GluC-1 (Os02g0453600)	133
GluD	Glutelin D-1	AAGACAGAGCGACCAAGCTC	ATGTGCAACACTAGCCGGAA	[b]GluD-1 (Os02g0249000)	100
**ER-Stress Response**					
OsBiP1	Binding protein 1	GGACTCAATCTCAACACGGAC	AGGACATCAGCAAGGACAAC	OsBiP1 (Os02g0115900)	107
OsBiP2	Binding protein 2	GAGACCAAAGACATCTTGCTG	GTCTGGTGATCCTCGTAGGT	OsBiP2 (Os03g0710500)	149
OsSar1a	GTP-binding protein Sar1a	AGTGTTGTCCGCAAGATGGG	CGCTGGGCAGAGTATGCAAG	OsSar1a (Os01g0338000)	101
OsSar1b	GTP-binding protein Sar1b	GCAAGATGGGCTATGGGGA	TGGTAAGGTGAAACAGGAGTATGAAC	OsSar1b (Os12g0560300)	124
OsSar1c	GTP-binding protein Sar1c	GCGTCGTCCGCAAGATG	AGGAGAGTTGATAAACAGAACCAGAG	OsSar1c (Os01g0254000)	163
CNX	Calreticulin precursor protein	TCGACAACCCCAACTACAAAG	ATCTCAATCCCAATAGCGGC	CNX (Os04g0402100)	112
PDIL1;1	Protein disulfide isomerase like 1-1	AACGATGTGCCAAGCGAGTTCGAT	TTAGAGCTCATCCTTGAGAGGCTC	PDIL1;1 (Os11g0199200)	201
PDIL2;3	Protein disulfide isomerase like 2-3	ATAAGAGGATTTCCAACTATTAAG	TGCTCCTTGATAATCTACTG	PDIL2;3 (Os09g0451500)	66

Supplementary Table S2. List of primers (continued).

Symbol	Name	Forward primer	Reverse primer	Target genes (Locus ID)	Product size
**Starch Synthesis**					
OsAGPS1	ADP-Glucose Pyrophosphorylase Small Subunit 1	AACCTCGATACTTGCCTCCA	CTTTCTTGTCCGTCTCCGTC	OsAGPS1 (Os09g0298200)	197
OsAGPL2	ADP-Glucose Pyrophosphorylase Large Subunit 2	AATCCTCCCGAAAGAATTATGCTG	TCAATAAGCCTGTAACATCCTCCA	OsAGPL2 (Os01g0633100)	141
OsAGPL3	ADP-Glucose Pyrophosphorylase Large Subunit 3	ATCTTTCCTCCTCTTTCCCTG	AACCAAGACACATCCTCCTC	OsAGPL3 (Os05g0580000)	271
OsAGPL4	ADP-Glucose Pyrophosphorylase Large Subunit 4	CAAATCTTGCTCTCACAGATCAG	AACGGACACCAATAACAGAG	OsAGPL4 (Os07g0243200)	185
OsSBE1	Starch-Branching Enzyme 1	GTCTCACCTCCTCTTCCTCC	ACCTCCTCCACAACAGTCAC	OsSBE1 (Os06g0726400)	210
OsSBE3	Starch-Branching Enzyme 3	ACTTCACTGCCGATTGTTCAC	CTTGGTGTTCTCATTCCGCT	OsSBE3 (Os02g0528200)	104
OsGBSS (WX1)	Granule-bound starch synthase	GGTGAGGATGTTGTGTTCGT	CTGGTAGGAGATGTTGTGGA	OsGBSS (WX1) (Os06g0133000)	134
OsSSII-3	Soluble Starch Synthase 2-3	CTTCCATCCGTAGACAAGCC	ATCAGCCCAGTCATCATCGT	OsSSII-3 (Os06g0229800)	293
**Internal Reference**					
UBI	Ubiquitin	TGGTCAGTAATCAGCCAGTTTGG	GCACCACAAATACTTGACGAACAG	UBI (Os02g0161900)	81

[a] represents gene name mentioned in Saito et al. (2012).

[b] represents gene name mentioned in Kawakatsu et al. (2008).
